# Atom devices based on single dopants in silicon nanostructures

**DOI:** 10.1186/1556-276X-6-479

**Published:** 2011-07-29

**Authors:** Daniel Moraru, Arief Udhiarto, Miftahul Anwar, Roland Nowak, Ryszard Jablonski, Earfan Hamid, Juli Cha Tarido, Takeshi Mizuno, Michiharu Tabe

**Affiliations:** 1 Research Institute of Electronics, Shizuoka University, 3-5-1 Johoku, Nakaku, Hamamatsu, 432-8011, Japan; 2 Division of Sensors and Measuring Systems, Warsaw University of Technology, Sw. A Boboli 8, 02-525 Warsaw, Poland

**Keywords:** single-dopant electronics, single-electron tunneling, double-donor systems, multiple-donor systems, photon, Kelvin probe force microscopy, silicon-on-insulator field-effect transistor

## Abstract

Silicon field-effect transistors have now reached gate lengths of only a few tens of nanometers, containing a countable number of dopants in the channel. Such technological trend brought us to a research stage on devices working with one or a few dopant atoms. In this work, we review our most recent studies on key *atom *devices with fundamental structures of silicon-on-insulator MOSFETs, such as single-dopant transistors, preliminary memory devices, single-electron turnstile devices and photonic devices, in which electron tunneling mediated by single dopant atoms is the essential transport mechanism. Furthermore, observation of individual dopant potential in the channel by Kelvin probe force microscopy is also presented. These results may pave the way for the development of a new device technology, i.e., single-dopant atom electronics.

## Introduction

After demonstration of the first semiconductor transistor, it was soon realized that semiconductors require doping with impurity atoms for achieving useful functionalities [1,2]. Dopants have played a key role in the fast-paced development of the electronics industry, dominantly based on silicon. As predicted by Moore's law in the 1960s [3], silicon transistors were expected to go through a miniaturization process, and this trend has been pursued for nearly half a century now. The transistor's gate length is now only several tens of nanometers, and it will be further downscaled to the 22-nm node and beyond [4]. This miniaturization faces many technological and fundamental challenges, among which a key problem is the discrete dopant distribution in the device channel [5]. Each individual dopant, having basically an uncontrolled position in the channel, significantly affects device characteristics [5,6], leading to device-to-device variability. It was shown that controlled positioning of dopants by single-ion implantation in the device channel can reduce threshold voltage variability in metal-oxide-semiconductor field-effect transistors (MOSFETs) [7].

On the other hand, technological progress offers a unique opportunity, i.e., electrical access to individual dopant atoms in nanometer-scale devices. Properties of dopant atoms that have been so far inferred from measurements of bulk materials, containing a large number of dopants, can now be associated to a specific dopant atom through direct measurements.

**Figure 1:**
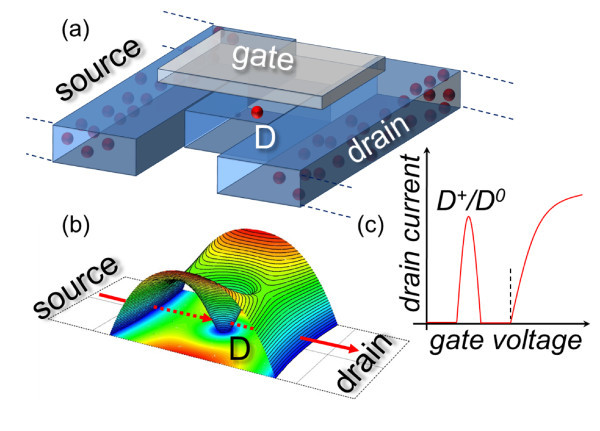
**Operation of a single dopant transistor**. (a) Schematic illustration of a single-donor transistor, in this case a transistor that contains one donor in its nanoscale channel. (b) The conduction path donor mediates single-electron tunneling from source to drain, giving rise to a current peak in the low-temperature transfer characteristics (c).

Recently, breakthrough results indicate the possibility of individually addressing dopants in silicon, as illustrated in Figure 1. When the dopant is located in a nanoscale-channel field-effect transistor (FET), single-electron tunneling via the dopant-induced quantum dot (QD) gives rise to measurable currents. Results illustrating this operation mode have been obtained at cryogenic or low temperature in transistors containing in their channel one or only a few dopant atoms. Single-electron tunneling spectroscopy of arsenic (As) donors, located in the edges of FinFET channels, was performed at cryogenic temperatures [8-10]. Acceptors, such as boron (B), were also directly identified in low-temperature transport characteristics of silicon-on-insulator (SOI) FETs [11,12]. Individual dopants can be accessed even in dopant-rich environments, where the channel contains more than only one isolated dopant atom [13]. In order to observe individual donor and/or acceptor impurities, scanning probe techniques are typically used, among which Kelvin probe force microscopy (KFM) allowed mapping of dopants in channels of Si nanodevices under normal operation conditions [14,15].

These findings accelerated research on individual dopant atoms in silicon nanostructures, as well as on interactions between dopants and the surrounding environment. Donor interface coupling has been intensively studied mostly from a theoretical approach [16-18], mainly for quantum computing [19,20], which involves transfer of an electron between a donor and a nearby interface. Furthermore, it has been predicted that dopant properties are significantly different in nanostructures due to effects such as dielectric confinement and quantum confinement [21]. It was found that the activation energy of dopants is enhanced in nanostructures, leading to reduced doping efficiency in doped Si nanowires [22]. This opens, however, an additional opportunity in terms of physics, since it may be possible to tune the properties of dopants by a suitable design of the nanoscale environment.

These developments justify an increasing interest in studies of single dopants in nanoscale Si transistors. Such studies may eventually enable us to develop an entire field of electronics, *single-dopant electronics*, in which the basic operation mode will be single-electron tunneling mediated by an individual dopant.

In this paper, we outline some of our recent results, focused on isolating single dopant features in electrical characteristics of nanoscale phosphorus-doped channel SOI-FETs. First, we will show that single dopants can be electrically addressed even in devices that contain more than just one isolated dopant in the channel [13]. For more advanced device functions, coupling between discrete dopants is expected to play a key role. Systems in which we identified two coupled donors dominating the characteristics will be described [23]. We will also address other application possibilities, such as single-electron turnstiles, that can be conceived using more complex donor arrays [24-27]. Additional functionalities can be expected when photons are incorporated in device operation [28]; as a first step, we will show results that indicate trapping of single photon-generated electrons by individual donors in an FET channel. Direct observation of dopant potentials in device channels using a Kelvin probe force microscopy technique [14,15,29] will also be presented. An overview of possible research directions in the field of single-dopant electronics will be given before the summary.

## Single-dopant transistors

Signatures of single-dopant atoms mediating the current in FETs have been found recently, in devices that contain a limited number of dopants in the channel. In FinFETs with fine control gates and nominally undoped channels, it is likely that a few donors diffuse from the source and drain heavily doped regions into the channel [8]. At very low temperatures, single-electron tunneling currents via these donors can be measured [8,9]. Single-hole tunneling via an isolated acceptor impurity was also identified in transport characteristics of nanoscale SOI-FETs with a small number of B atoms implanted in the channel by low-dose ion implantation [11,12]. These studies showed direct transport via isolated donor or acceptor atoms in nanoscale channels, but with a small number of dopants randomly located in the channel. Due to this, only a fraction of the measured devices exhibited transport through dopants [9,11].

**Figure 2:**
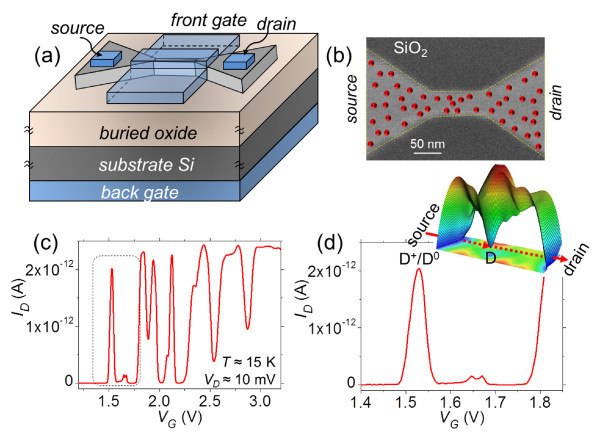
**Single dopants in dopant-rich environment**. (a) Device structure of a phosphorus-doped SOI-FET with the channel containing several donors. (b) SEM image of the channel area with an illustration of random donor arrangement. (c) An example of device characteristics with a single isolated first peak, indicating single-electron tunneling via a donor QD even in a donor-rich channel. (d) Zoom in on the first peak (inset: single-electron tunneling via a donor in a donor-rich channel).

Devices with higher doping concentration are attractive because the presence of dopants in the channel can be confirmed. The problem is understanding if, in such dopant-rich environments (channels that contain more than just one isolated dopant), signatures of transport via individual dopant atoms can still be observed. For that purpose, we investigated devices having a structure as shown in Figure 2a: SOI-FETs with the channel patterned by an electron beam lithography technique to have a width of about 50 nm and a length between 20 and 150 nm [see Figure 2b]. Top Si layer has a final thickness of only 10 nm and is doped uniformly with phosphorus to a concentration *N_D _*≅ 1 × 10^18 ^cm^-3 ^(as estimated from secondary ion mass spectrometry of reference samples). A simple estimation would give a number of 10 to approximately 75 dopant atoms in the device channel, depending on the channel dimensions.

We measured the drain current-gate voltage (*I_D_*-*V_G_*) characteristics of SOI-FETs at low temperatures (approximately 15 K) and low source-drain biases (*V_D _*= 5-10 mV). All devices exhibit irregular current peaks, as seen in Figure 2c, which can be ascribed to single-electron tunneling mediated by QDs. In our devices, QDs are introduced by the channel donors. The peaks are not periodic in *V_G_*, which indicates that the QDs are not metallic and that different QDs may be responsible for different current peaks. It is thus natural to conclude that individual peaks can be ascribed to one or a few discrete donors that are active in transport. At the initial stages of transport, i.e., when the lowest channel states are aligned with source Fermi level, it becomes easier to identify transport via discrete donors because the channel is mostly depleted of free electrons. This is why we focus on the first observable current peaks (*I_D _*> 10 fA), which correspond to single-electron tunneling via states below conduction band, i.e., via donors, as illustrated in Figure 2d.

We found that most devices have complex first current peaks, with several subpeaks incorporated in the peak envelope. This can be understood as a simultaneous contribution of several QDs to transport [30]. This effect is generally more pronounced for devices with longer channels, for which the probability of finding a larger number of donors with comparable energies, i.e., simultaneously contributing to transport, is enhanced. For short-channel devices, however, it is possible to identify characteristics containing a single isolated first current peak, without inflections (as in Figure 2). In these devices, a single donor controls single-electron tunneling transport for the *V_G _*range around the peak. This has been supported by simulations [13] considering a simple model in which individual donor Coulomb potentials are superimposed in order to form the overall potential landscape in the channel [31]. For a statistical number of donor arrangements, we found a high probability to identify a single donor as the origin of the first current peak [13]. These findings open new possibilities for the study of single-dopant devices because an individual donor can work as conduction path even in channels containing more than only one donor. The conduction path donor is an equivalent QD with charge occupancy limited to one electron at practical temperatures.

Ideally, a single-dopant transistor contains one dopant precisely located in the center of a nanoscale channel. Significant progress has been realized in the direction of dopant positioning by either top-down approaches, such as single-ion implantation [7,32-34], or bottom-up approaches [35,36]. An alternative way to control the location of the active dopant would be to take advantage of the effects of nanopatterning the channel; by imposing specific patterns on randomly doped channels, the probability of successfully isolating a single dopant is enhanced. Results on this topic will be presented elsewhere.

The results described in this section provide the grounds for development of a key device, a single-dopant transistor, in which the operation principle is single-electron tunneling through a QD which is not lithographically defined, but naturally created by an individual dopant atom. This can be seen as the building block for more advanced functionalities.

## Coupled-donor systems

A double-donor system in Si has been considered the basic unit for quantum computing, either based on spin states [19] or charge states [20]. We study double-donor systems from a more practical approach, i.e., to demonstrate functions such as a dopant-based memory, with one donor as a sensor (conduction path) and another donor as a memory node (trap). If such a system could be identified, it would become possible to design memories in which sensing is done by a single-electron tunneling current via one donor atom, while storage is ensured by an individual donor working as a memory node.

**Figure 3:**
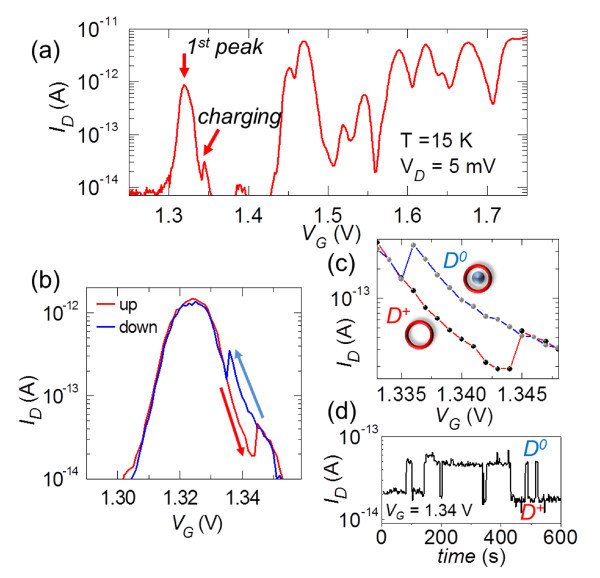
**Single-electron transfer between two donors**. (a) Low-temperature *I_D_-V_G_* characteristics showing a single-donor current peak used as a sensor for detecting charging and discharging of a neighboring donor. (b)-(c) Charging and discharging are sensed as abrupt jumps of the current (producing a hysteresis between up-ramping and down-ramping curves) and (d) as RTS in the time-domain measurements.

We have focused on identifying signatures of such a double-donor system among our devices, which contain donors randomly distributed in the channel [23]. The first target was to select devices that exhibit characteristics with a single isolated first peak. As also described in the previous section, a single isolated current peak can be found when only one donor-induced QD controls electron transport, i.e., when the single-electron tunneling conduction path contains only one donor [13]. When the gate voltage aligns the conduction path donor's energy with the source/drain Fermi level, conduction through the device starts. Characteristics for such a device are shown in Figure 3a.

We planned to use this conduction path donor as a sensor for detecting changes of the charge states of nearby donors. For that, we measured *I_D_*-*V_G _*characteristics by up-ramping and consecutively down-ramping *V_G _*around the first peak, as shown in Figure 3b. For most devices, changes in the background charges could not be observed. However, for some devices, as the one shown in Figure 3, we identified abrupt current jumps reflecting sudden changes in potential due to a charging or discharging event. Charging and discharging, observed in consecutive up-ramping and down-ramping sweeps, gives rise to a hysteresis in the characteristics [zoomed-in in Figure 3c]. This suggests that, within the hysteresis *V_G _*region, the charge state of the trap is different, depending on the sweeping direction. *I*_D _time measurements [see Figure 3d] show two-level random telegraph signals (RTS), suggesting a two-level trap. In our device, it is most natural to assume that the trap is a donor, either ionized (D^+^) or neutralized (D^0^). This is because the number of donors in the channel is larger than the estimated number of interface defects. Furthermore, from our previous studies [24] comparing doped and undoped channel FETs, it is evident that most of the features (irregular peaks) observed in the measured characteristics are due to the channel donors. Our further analysis [23] reveals that the trap-donor is closer to the front interface as compared with the conduction path donor. We thus identified devices in which two donors work as a sensor and as a memory node, respectively. This allows further investigation of the physics behind single-electron transfer within double-donor systems.

The particular feature of a two-donor system, compared to other single-electron memory proposals [37,38], is that each donor can practically only accommodate one electron. Although a second electron could be added to a donor, the energy level for this state (*D*^-^) resides close to the conduction band edge [39], and it is not expected to be observed under our measurement conditions. In our devices, donors are embedded into a thin Si layer (10 nm) and, in consequence, reside close to the Si/SiO_2 _interface. For such donors, an increasing electric field shifts the electron wavefunction towards the interface, while still maintaining the electron localization around the donor [16,17,9]. Localization length at the interface is gradually increasing with electric field; in our devices, as *V*_G _is increased, a donor closer to the surface would expand its potential at the interface. This means that the cross-sectional area of the donor QD, seen from the gate, is gradually increasing. In this situation, we suggested that the donor-gate capacitance should be *V*_G _dependent, which allowed us to reproduce in simulation single-electron transfer between the two single-donor QDs [23]. Using this simulation, we investigated the effects of different donor arrangements on the hysteresis width [23]. With further progress in dopant engineering, a controlled design of dopant-based single-electron memory devices, working on a principle as described here, could become feasible [40].

A wide range of applications can be envisaged when we consider more complex donor arrangements. We demonstrated that an array of several donors, simultaneously working within a single-electron tunneling conduction path, can allow a time-controlled single-electron transfer between source and drain [24,25]. In phosphorus-doped nanowire-FETs, an ac gate voltage can change the charge state of the system by exactly one electron. An electron enters the system during the high level of the pulse and leaves the system during the low level. Injection occurs from the source, while extraction occurs to the drain, which gives rise to a single-electron/cycle transfer between the two electrodes. This operation is similar to single-electron turnstile devices proposed with metallic QDs [41] or with semiconductor QDs [42,43], with the key difference that in our devices QDs are dopant atoms. From simulation studies [26,27], we found that the natural inhomogeneity of device parameters (mainly donor-gate capacitances) plays an important role in single-electron turnstile operation.

In short, various applications can be designed using charge states of coupled donors, suggesting that there is a rich environment for further research and development of functionalities downscaled to the level of discrete donors.

## Photon effects in doped channel FETs

Functionalities presented so far rely on single-electron tunneling via one donor or coupled donors in dark conditions, i.e., without photon illumination. However, the range of applications for donor-based systems can be drastically enhanced by purposely incorporating photon effects. Based on the interaction between photons and dopants, dopant-based optoelectronic devices could be developed.

The effects of photon illumination on semiconductor devices have been studied for a long time, mainly for developing high-speed photodetectors [44] or solar cells [45]. For FET devices, when photons are irradiated on the channel, a fraction of incident photons will be absorbed in the device active region. By absorption of a photon, an electron-hole pair is generated, and carriers may either contribute to conduction, recombine with each other, or become trapped in available traps. Significant research has been done on demonstrating trapping of photo-generated carriers in QDs [46,47]. It was found that QD arrays may work as a building block for single-photon detection, involving trapping of elementary charges in a QD and sensing it with a current flowing as a percolation path in the channel. Such devices have low quantum detection efficiencies, but demonstrate that single photons can play an active role in the trapping of single carriers.

**Figure 4:**
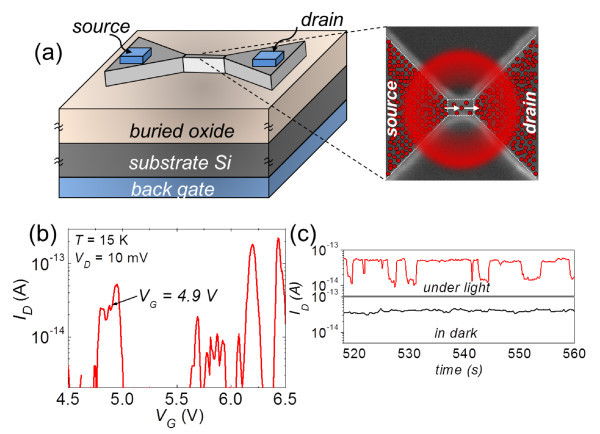
**Photon-generated electron trapping in donor arrays**. (a) Device structure of a back-gated SOI-FET for light illumination measurements. With *V_G _*set on the first observable current peak (b), effects of photon absorption in the nanoscale channel can be observed as RTS in the time-domain measurements (c).

A similar mechanism could be expected when the QD array is replaced by an array of donors. In order to clarify this point, we investigated the effects of (visible) light irradiation on doped-channel FETs without a front gate. For these devices, the substrate Si was used as a back gate, allowing an operation similar to that of front-gate devices. The basic device structure is shown in Figure 4a. We first measured the low-temperature (approximately 15 K) *I_D_*-*V_G _*characteristics, as shown in Figure 4b, under conditions of visible light illumination (*λ *= 550 nm) with low incident flux. The characteristics exhibit irregular current peaks, similarly to front-gate devices. We focused on the first observable peak that can be ascribed to single-electron tunneling transport via lowest-energy donors in the channel [13]. We aimed at clarifying whether photon-generated carriers can be trapped or not in the remaining available donors.

A basic result on this point is shown in Figure 4c. We measured the time-domain characteristics in the dark with *V_G _*set on the first peak and observed no significant current changes (lower panel). This indicates that, in the dark, background charges do not naturally fluctuate significantly. Under light illumination, however, the characteristics are strikingly different, exhibiting an RTS pattern (upper panel). This proves that photon-induced carriers remain trapped in the channel for sufficient time to be sensed in our measurement. In most devices measured, the RTS has mainly two levels, which suggests that only one trap is responsible for the observed current switching. It is again natural to ascribe the trap to a donor located in the device channel. We identified the charge states of the trap-donor by comparison with *I_D_*-*V_G _*characteristics, and we found that the frequency of single-electron trapping is directly proportional to the photon flux [28]. These results suggest the possibility of incorporating photons into single-dopant device operation, opening a rich field of study on the interaction between individual dopants in silicon and their electromagnetic environment.

## Single-dopant observation

In order to fully understand the properties of dopant-based devices, it is necessary to directly observe the dopant distribution in the device channel. For this purpose, several techniques have been proposed, such as scanning tunneling microscopy (STM) and scanning capacitance microscopy (SCM). Individual dopants were successfully observed using STM techniques on samples with a specific surface treatment [35,48,49]. STM, however, relies on measuring tunneling currents between a metallic tip and the sample, and it can only detect point charges located in the topmost few layers. SCM is based on evaluating the capacitance between a tip and the sample, allowing for subsequent extraction of dopant profiles [50,51]. This technique is, however, limited by the tip and sample quality, which strongly affect the measured capacitance values.

The main issue with mapping dopants in FET channels is having the ability to measure signals coming from dopants located within a thin layer of Si, covered by a thin SiO_2 _film, as the case of SOI-FETs. The most suitable way to do that is to detect the long-range electrostatic force created by an ionized dopant. This is the basic principle of operation for a technique called Kelvin probe force microscopy (KFM) [52]. Basically, KFM allows measurement of contact potential difference between a metallic tip and the sample surface [52], with additional contributions from dipole formation near the surface of the semiconductor sample [53].

**Figure 5:**
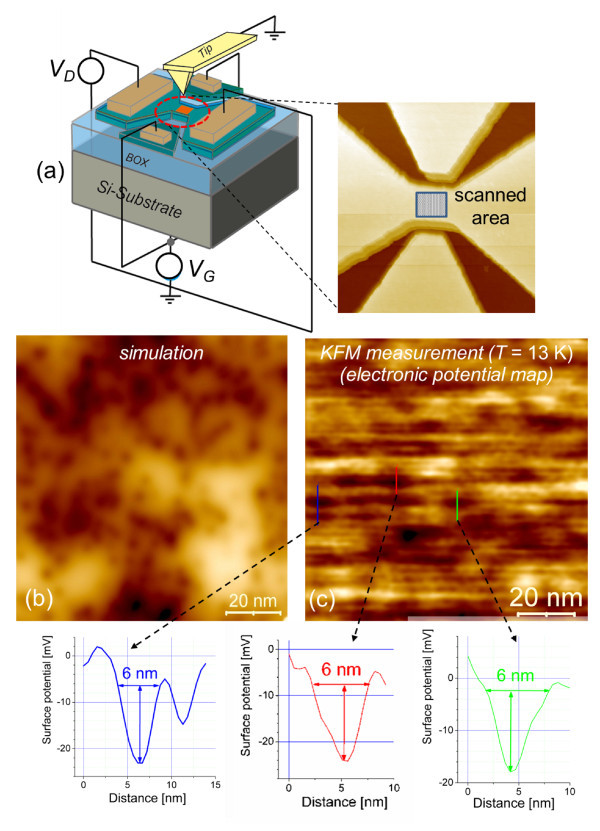
**KFM observation of discrete dopants in device channel**. (a) Setup for LT-KFM measurements, showing in the inset the topography of the measured channel area. (b) Simulated surface electronic potential map due to ionized P donors in a thin Si layer. (c) Measured electronic potential maps at the surface of P-doped SOI-FETs (lower panels are line profiles through some of the dark spots, i.e., regions of lower electronic potential).

We have utilized a special setup of KFM, which allows us to measure FETs fabricated on SOI wafers, at low temperatures (approximately 13 K), with the possibility of fully using external biases [see Figure 5a]. Applying an appropriate bias to the devices can induce depletion of the channel of free carriers, leaving behind immobile charges, i.e., ionized dopants. Depletion can be achieved more readily at low temperatures, at which intrinsic carrier concentration is negligible, allowing for a measurement of unscreened ionized dopants [15]. Expected results are shown in Figure 5b from a simulation of electronic potential due to a random distribution of phosphorus (P) donors in a thin Si layer. Using our low-temperature (LT)-KFM technique, we measured the discrete distribution of P donors in the channel of thin SOI-FETs [14], as shown in Figure 5c as electronic potential maps. A darker contrast indicates lower electronic potential, consistent with the presence of a positive phosphorus ion (P^+^). The spatial extension of the dark spots is typically below 10 nm, while the potential depth is on average a few tens of meV, as observed from insets in Figure 5c. These characteristics are in good agreement with properties of individual P donors, such as Bohr radius (*r*_B _≅ 2.5 nm) and ground state energy (*E*_0 _= 44 meV). Acceptor impurities, such as boron (B), could also be observed when measuring weakly doped p-type Si samples [14]. These results prove the potential of the KFM technique to resolve the distribution of dopants in FET channels at single-dopant level. Our recent studies also demonstrate the ability of LT-KFM to detect different charge states of isolated or clustered donors [29].

## Summary and conclusions

As briefly outlined, dopants in semiconductors provide a wide range of applications based on manipulation of elementary charges and dopant states. Koenraad and Flatté [54] have recently given an extended review on single dopants in semiconductors, covering also dopant-based spintronics and dopants as nonclassical light sources.

**Figure 6:**
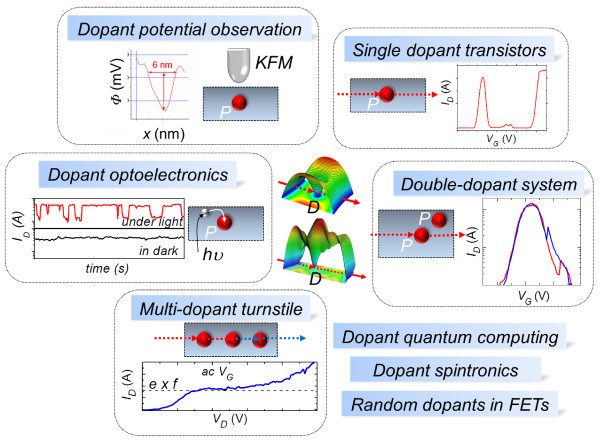
**Schematic overview of research directions on individual dopants or coupled dopants in semiconductor devices**. Individual donors, either isolated or in a many-donor channel, can be at the basis of a variety of applications: single dopant transistors, memory or turnstile devices in double- or multiple-dopant systems, and dopant optoelectronic devices based on photon-dopant interaction. Complementary directions can be towards dopant quantum computing or spintronics applications, as well as towards addressing the issue of random dopants in FETs. KFM can provide a way to directly observe effects associated with discrete dopants in operating devices.

A simple overview on the possible directions of research involving individual dopants, either isolated or in dopant-rich channels, is shown in Figure 6. Single-dopant transistors can become attractive candidates for applications involving electron transport at atomic level. Studies of coupled donors in nanostructures may reveal more complex properties that arise from interactions among donors and between donors and interfaces. Dopant-based optoelectronic devices can be conceived based on studies of photon illumination on dopant arrays. In addition, active research in controlling and monitoring of discrete dopants in working devices will support the steady development of *single-dopant electronics*. Quantum computing, dopant spintronics, and challenges related to dopant distribution in conventional FETs will continue as important components for understanding individual dopant properties. All these directions will converge in creating a rich research environment able to push the silicon-based electronics industry beyond the limits of the Moore's law and into atomic scale functionalities. It is also essential to note that the properties of the dopants significantly change when the donors are located in nanostructures, compared to bulk. A basic finding is that dopants embedded in nanowires may have an enhanced binding energy due to dielectric or quantum confinement [21]. We suggest that this effect can play a key role in the development of single-dopant devices operating at higher temperatures and further studies may reveal the guidelines to utilize this effect up to room temperature.

In this paper, we briefly outlined some of the key functions of dopant atom devices, such as single-electron tunneling via a donor in donor-rich devices, single-electron transfer between two donors, time-controlled transport through donor arrays, effects of photons on individual donors, as well as direct observation of dopant potentials. With further progress under way in dopant control and observation, coupled with new findings from theoretical treatment of dopants in nanostructures, the field of *single-dopant electronics *is expected to unfold into a rich area of research for ultimately miniaturized devices and beyond.

## Competing interests

The authors declare that they have no competing interests.

## Authors' contributions

DM carried out experiments on single-dopant transistors and single-electron turnstiles and contributed to other experiments. AU carried out experiments on photon effects on doped FETs. MA and RN performed experiments on KFM observation of doped FET channels, based on discussions with RJ and MT. EH and JCT measured FETs at low temperature for identifying single-electron transfer between two donors. TM provided technical assistance in device fabrication and experiments. MT conceived and supervised the experiments and discussed the results. DM and MT co-wrote the manuscript. All authors read and approved the final manuscript.
